# Effect of high‐intensity interval training on cardiometabolic component risks in persons with paraplegia: Results of a randomized controlled trial

**DOI:** 10.1113/EP091803

**Published:** 2024-06-24

**Authors:** Matthew Farrow, Jennifer Maher, Rachel Deere, Bruno Spellanzon, Sean Williams, Dylan Thompson, James L. J. Bilzon

**Affiliations:** ^1^ Department for Health University of Bath Bath UK; ^2^ Centre for Nutrition and Exercise Metabolism (CNEM) University of Bath Bath UK; ^3^ Department of Physical Medicine & Rehabilitation Ohio State University Columbus Ohio USA; ^4^ Centre for Trials Research, College of Biomedical & Life Sciences Cardiff University Cardiff UK

**Keywords:** cardiovascular disease, exercise, spinal cord injury

## Abstract

**Abstract:**

The aim of this work is to determine the effect of upper‐body high intensity interval training (HIIT) on cardiometabolic risks in individuals with chronic paraplegia. Twenty‐seven individuals (14 females, 13 males, mean ± SD age: 46 ± 9 years) with chronic paraplegia (spinal cord injury between T2 and L5 >1‐year post‐injury) took part in a randomized controlled trial and were included in the final analysis. Participants in the HIIT group (*n* = 18) performed ∼30 min of arm crank exercise (60 s intervals at 80%–90% peak heart rate) four times per week, for 6 weeks. Participants in the control (CON) group (*n* = 9) were asked to maintain their habitual diet and physical activity patterns over the study period. Outcome measures were taken at baseline and follow‐up. The primary outcome measures were fasting insulin, peak power output (PPO) and peak aerobic capacity (${\dot{V}}_{O_{2}\mathrm{peak}}$). Secondary outcome measures included body composition, postprandial glycaemic control, fasting blood lipids, inflammatory biomarkers and resting blood pressure. Differences between groups were assessed by ANCOVA, using baseline values as a covariate. PPO was higher in the HIIT (101 W, 97–106) compared to the CON (90 W, 83–96) group at follow‐up (*P* = 0.006). There were no differences in fasting insulin (*P* = 0.415) or relative ${\dot{V}}_{O_{2}\mathrm{peak}}$ (*P* = 0.417). Postprandial Matsuda insulin sensitivity index (ISI_Matsuda_) was higher in the HIIT (5.42, 4.69–6.15) compared to the CON (3.75, 2.46–5.04) group at follow‐up (*P* = 0.036). Six weeks of upper‐body HIIT increased PPO and ISI_Matsuda_, with no other beneficial effect on cardiometabolic component risks in persons with chronic paraplegia.

**Highlights:**

- **What is the central question of this study?**
  What is the effect of upper‐body high intensity interval training (HIIT) on cardiometabolic component risks in individuals with chronic paraplegia?
- **What is the main finding and its importance?**
  Six weeks of upper‐body HIIT increased PPO and improved insulin sensitivity, but had no beneficial effect on other cardiometabolic component risks in persons with chronic paraplegia. The large effect size observed for insulin sensitivity may be important in terms of reducing the risk of type‐2 diabetes in this population.

## 1 INTRODUCTION

Globally, it is estimated that there are ∼2 million people living with a spinal cord injury (SCI) (Lee et al., 2014). In the UK, one in three deaths in persons who have sustained a traumatic SCI and survived the first year (i.e., chronic SCI) can be attributed to cardiovascular disease (CVD). When adjusted for age and sex, mortality rates associated with CVD are three times greater among people with SCI, compared to the non‐injured population (Savic et al., 2017). Persons with a chronic SCI have a high prevalence of component risks associated with CVD, including impaired glucose tolerance (Cragg et al., 2013), central adiposity (Edwards et al., 2008), chronic inflammation (Wang et al., 2007) and dyslipidaemia (Gilbert et al., 2014). Therefore, therapeutic solutions are required for this population to reduce their risk of developing CVD.

Despite the well‐established link between physical activity and cardiovascular disease in the non‐injured population, the majority of persons with chronic SCI are habitually inactive, performing little to no moderate‐to‐vigorous physical activity (Buchholz et al., 2009; Nightingale, Williams et al., 2017). The latest SCI exercise guidelines differ in the volume of exercise/physical activity recommended to reduce cardiovascular disease (90 min of moderate‐to‐vigorous exercise per week) (Ginis et al., 2018) and cardiometabolic disease (150 min of moderate intensity physical activity) risk (Nash et al., 2018). However, a randomized controlled trial found that performing 4 × 45 min per week of moderate‐intensity aerobic exercise was sufficient to improve cardiorespiratory fitness and fasting insulin sensitivity, but no changes were observed in lipid profile, body composition or postprandial glycaemic control, amongst physically inactive individuals with chronic SCI (Nightingale, Walhin et al., 2017). This quantity of exercise (180 min/week) is higher than the physical activity guidelines for SCI and non‐injured humans (150 min/week), and suggests that a higher intensity, or greater volume of exercise may be required to achieve further cardiometabolic health benefits for this population. Given the complex barriers to exercise participation this population faces (Kehn & Kroll, 2009), promoting a higher volume of exercise seems unrealistic.

Instead, a viable solution may be to maximize the intensity of exercise performed, by prescribing high‐intensity interval training (HIIT). This form of exercise can be generally characterized as involving short intervals eliciting ≥80% (but often 85%–95%) of maximum heart rate (MacInnis & Gibala, 2017), and is an established training method to improve insulin sensitivity, blood pressure and body composition in individuals at risk of CVD (Campbell et al., 2019). Several meta‐analyses have also reported superior effects of HIIT in comparison to moderate‐intensity continuous training (MICT) for cardiorespiratory fitness (Weston et al., 2014), insulin resistance (Jelleyman et al., 2015) and diastolic blood pressure (Ramos et al., 2015) in non‐injured humans. Meta‐analyses have also reported that HIIT is equally effective as MICT at improving the lipid profile (Wood et al., 2019) and inflammatory markers (Khalafi & Symonds, 2020) in non‐injured humans.

There has been growing interest in prescribing HIIT for persons with SCI since Nightingale, Metcalfe et al. (2017) proposed a plausible biological mechanism for improving cardiometabolic health outcomes in this population. Of particular note, a randomized controlled trial determined that 5 weeks of upper‐body sprint interval training (3 × 20 s ‘all‐out’ sprints) was equally as effective as 25 min of MICT (45% peak power output) for improving peak power output in individuals with sub‐acute SCI (McLeod et al., 2020). Additionally, a pilot study in persons (*n* = 7) with chronic SCI found that 6 weeks of upper‐body HIIT was equally effective as MICT for improving insulin sensitivity and aerobic fitness, despite a reduced weekly training volume (40 min vs. 90 min) (Graham et al., 2019). However, to date, there are no randomized controlled trials that have assessed the effect of upper‐body HIIT on a range of cardiometabolic component risks in persons with SCI.

The purpose of this randomized controlled trial was therefore to determine the effect of an upper‐body HIIT intervention on cardiometabolic component risks in persons with chronic paraplegia. Participants were randomly assigned using a 2:1 allocation to a 6‐week home‐based HIIT intervention, or a control group that maintained their normal lifestyle over the study period, chosen to reflect the habitually low physical activity levels in this population. The primary outcome measures were fasting insulin, peak aerobic capacity, and peak power output. We hypothesized that fasting insulin concentrations would be reduced and both peak cardiorespiratory capacity and power output would be increased following 6 weeks of HIIT compared to the control group. Other secondary and exploratory outcome measurement categories included: (i) body composition, (ii) postprandial glycaemic control, (iii) lipid concentrations, (iv) inflammatory cytokines (including adipokines), (v) physical activity, (vi) energy intake, (vii) resting metabolic rate, and (viii) subjective perceptions of health and wellbeing.

## 2 METHODS

### 2.1 Study design

The protocol for this study was approved and published prior to study commencement (Farrow et al., 2021). This study was approved by the South‐West (Bristol) National Research Ethics Committee (REC reference number 20/SW/005, Version 2, dated 9 April 2020) and registered on ClinicalTrials.gov (ID: NCT04397250) on 21 May 2020. A randomized controlled trial was conducted, with participants randomly assigned to either a home‐based upper‐body HIIT intervention or a control group (CON). Participants in the HIIT group were asked to perform exercise (four sessions/week) for 6 weeks. Participants in the CON group were asked to maintain their habitual diet and physical activity routine during the 6‐week period. Baseline and follow‐up assessments for both groups were conducted at the DisAbility Sport & Health (DASH) laboratory at the University of Bath to determine the effectiveness of the intervention. A waiting‐list control group was utilized, with participants initially allocated to the CON group being offered the chance to take part in the intervention; however, no further measurements were taken from these participants. The study was conducted in accordance with ethical principles for studies involving human participants set out in the *Declaration of Helsinki*.

### 2.2 Recruitment

The primary recruitment pathway was the advertisement of the study to potentially eligible individuals on the databases of local NHS Research and Development offices (Duke of Cornwall Spinal Treatment Centre, National Spinal Injuries Centre, London Spinal Injury Centre, and Welsh Centre for Spinal Trauma). In addition, these centres displayed a recruitment poster in a publicly visible area. These offices sent out letters and participant information sheets to individuals on their database who were aged 18–65 years, with a SCI between the second thoracic (T2) and second lumbar (L2) vertebrae. For individuals from whom no communication was received within 1 month of the letter send‐out, one of the offices contacted the individuals via telephone or a follow‐up letter to enquire if they were interested in taking part. After this, there was no further direct contact with potential participants. Participants were invited to contact the research team at the University of Bath directly if they were interested in taking part in the study. In addition, participants were recruited using social media advertisements through non‐NHS charities and clinical partners. Interested potential participants were asked to contact the principal researcher for further information via email/telephone correspondence. The principal researcher emailed the participant information sheet and health screen and conducted a follow‐up phone call >48 h after the participant expressed their initial interest to fully explain what the trial entailed and answer any questions. Providing the potential participant indicated that they still wanted to take part in the study, the principal researcher scheduled the first visit. On the first visit, participants were asked to provide written informed consent.

### 2.3 Randomization

Eligible individuals were randomly assigned to either the HIIT or CON group. Randomization took place after the baseline visit. As recommended for trials involving small sample sizes (Altman & Bland, 2005), a minimization approach was used to balance groups for key characteristics (age, sex and time since injury) at baseline. This was performed by S.W., using a free program (sportsci.org/2010/wghminim.htm), with factors weighted equally and no random elements. An unequal allocation (2:1) was chosen to allow for a greater number of participants being assigned to the HIIT group, as it was expected that there would be large inter‐individual variation compared to the CON group. The research team and participants were not blinded to group assignments following the randomized allocation.

### 2.4 Participants and eligibility criteria

The participants recruited were aged between 18 and 65 years, with a chronic SCI (>1‐year post‐injury) between the T2 and L2, self‐reporting use of a wheelchair for >75% of their waking day, and weight stable (weight not changed by ≥3%) for the last 3 months. Individuals who self‐reported active medical issues such as pressure sores, urinary tract infections, cardiac disorders, cardiovascular contraindications for exercise testing (Goosey‐Tolfrey, 2007), or musculoskeletal complaints of the upper extremities were excluded. Individuals who self‐reported the use of type‐2 diabetes medication or drugs that effect glucose metabolism were excluded. This was checked on a case‐by‐case basis using the British National Formulary. Finally, any participants with plans to change their lifestyle (i.e., diet or physical activity) level during the study period were also excluded.

### 2.5 Laboratory assessments

**FIGURE 1:**
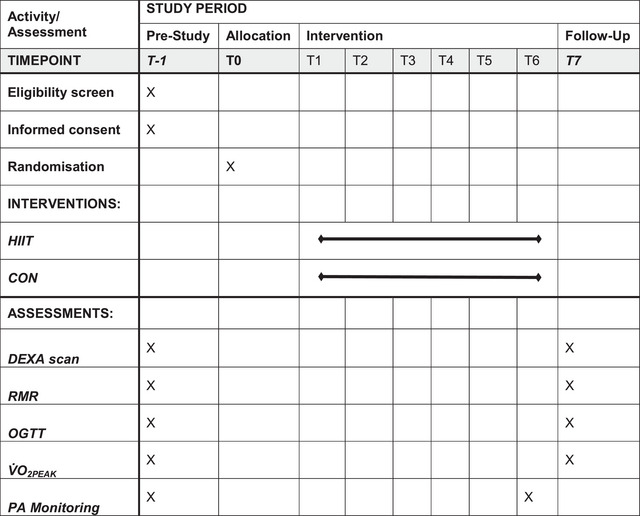
Standardized protocol items: Recommendations for Interventional Trials (SPIRIT) form. DEXA, dual‐energy X‐ray absorptiometry; OGTT, oral glucose tolerance test; PA, physical activity; RMR, resting metabolic rate; ${\dot{V}}_{O_{2}\mathrm{peak}}$, peak aerobic capacity.

The same experimental procedures were completed on both main trial days and are displayed in the Standardized Protocol Items: Recommendations for Interventional Trials (SPIRIT) form (Figure 1). Before each main trial day, participants were asked to refrain from performing any moderate or strenuous exercise in the 48‐h prior, and consuming alcohol and caffeine in the 24‐h prior. Participants arrived at the laboratory at the same time for both main trial days following an overnight fast (>10 h). Participants were also asked to mimic their food and drink intake in the 2 days before these visits using a non‐weighed food diary. Assessments were conducted during the follicular phase of the menstrual cycle (3–10 days after the onset of menses) for all eumenorrhoeic females taking part in the study.

#### 2.5.1 Body composition

Body mass was measured (to the nearest 0.1 kg) using platform wheelchair scales (Decto® BRW1000, Webb City, MO, USA), with the wheelchair and participants’ shoes weighed separately and subtracted from the total mass. Participants were asked to void prior to this measurement and remove all heavy clothing. For all body composition measurements, participants laid flat on a Dual‐energy X‐ray absorptiometry (DEXA) scanning table (Discovery, Hologic, Bedford, UK). Supine length was measured with participants lying flat on the bed, with their feet close together and arms at the side. Length was measured (to the nearest 0.5 cm) alongside the left‐hand side of the body, using a non‐elastic tape measure (Lufkin, Sparks, MD, USA). A wooden board was pressed against the feet, to achieve dorsal flexion. Waist and hip circumferences (to the nearest 0.1 cm) were taken in duplicate, using a non‐metallic tape measure. Waist circumference was measured at the end of normal expiration, at the mid‐way point between the lowest palpable rib and the iliac crest. Hip circumference was measured at the widest portion of the buttocks. The DEXA scan was performed with participants positioned in the middle of the scanning table, with their feet spaced evenly either side of the mid‐point of the body, arms placed mid‐prone with an equal gap to the trunk on both sides.

#### 2.5.2 Resting metabolic rate and blood pressure

Resting metabolic rate (RMR) was estimated via indirect calorimetry from 5‐min expired gas samples, collected into pre‐evacuated Douglas bags (Hans Rudolph, Shawnee, KS, USA) through a mouthpiece connected to a two‐way valve. Fractions of oxygen (O_2_) and (CO_2_) were measured using a paramagnetic O_2_ and infrared CO_2_ analyser (miniMP 5200, Servomex, Crowborough, UK), calibrated with known concentrations of gas (100% nitrogen, and 20% O_2_, 8% CO_2_) on the morning of testing. During each collection, ambient O_2_ and CO_2_ fractions were measured at close proximity to the participant to account for changes in an enclosed laboratory environment (Betts & Thompson, 2012). Expired fractions of O_2_ and CO_2_, total volume of expired gas (Harvard Apparatus, Edenbridge, UK), and expired gas temperature (model C, Edale Instruments, Cambridge, UK) were measured for each sample. All values were corrected to reflect atmospheric pressure and temperature during each collection. RMR was calculated using stoichiometric equations (Frayn, 1983), and taken as the average of three samples differing by ≤100 kcal/day. During the final 5 min of RMR, resting heart rate (Polar H10, Polar Electro, Vansbro, Sweden) was recorded every 30 s, and a mean taken. After the assessment of RMR, resting blood pressure was measured in triplicate using an automated blood pressure monitor, with 1 min rest in between each measurement.

#### 2.5.3 Blood sampling

A cannula was inserted into an antecubital vein, and a 20 mL blood sample drawn. Whole blood (and all blood samples during the oral glucose tolerance test) was dispensed into serum and plasma separation tubes. For serum, whole blood was placed in serum separation tubes and left to stand at room temperature for 15 min before centrifugation. For plasma, whole blood was placed in tubes coated with EDTA and centrifuged immediately. Samples were centrifuged at 4000 *g* for 10 min at 4°C, with 0.5 mL aliquots then obtained for serum and plasma. Aliquots were then immediately cooled on dry‐ice, before being stored in a −80°C freezer for long term storage. A small aliquot of the fasting blood sample was placed in a tube coated with EDTA to assess leukocyte differentials (SD‐300, Sysmex Ltd, Milton Keynes, UK).

#### 2.5.4 Oral glucose tolerance test (OGTT)

Participants consumed 113 mL of PolyCal (PolyCal, Nutricia Advanced Medical Nutrition, Trowbridge, UK) and 87 mL of water, within 5 min. Blood samples (5 mL) were drawn every 15‐min for the following 2 h. To ensure the cannula was kept patent, 0.9% NaCl was flushed through following each blood draw. During the last 30 min of the OGTT, participants completed the Wheelchair User's Shoulder Pain Index (WUSPI) (Curtis et al., 1995), the Short Form‐36 health survey (SF‐36) (Ware & Sherbourne, 1992), the Fatigue Severity Scale (FSS) (Anton et al., 2008), the Exercise Self‐efficacy Scale (ESES) (Kroll et al., 2007), and the Spinal Cord Independence Measure (SCIM) (Catz et al., 1997) questionnaires.

#### 2.5.5 Exercise testing

A sub‐maximal incremental exercise test was then performed on an electronically braked arm‐crank ergometer (Lode Angio, Groningen, Netherlands) consisting of four 3‐min stages, starting at 5 W, and increasing by either 10 or 15 W (depending on self‐reported fitness level). Participants were instructed to maintain a cadence of ∼75 rpm throughout. They wore a rubber facemask connected to a two‐way breathing value throughout, with expired gases (Douglas bag method, as previously described) and heart rate recorded in the final minute of each stage.

Participants were then given a small snack, before performing a maximal exercise test to determine peak aerobic capacity (${\dot{V}}_{O_{2}\mathrm{peak}}$). The ramp‐based protocol on an electronically braked arm‐crank ergometer began with a 2‐min warm‐up at 10 W before increasing by 1 W every 6 s. Participants wore a rubber facemask connected to a two‐way breathing valve, which was connected to a computerized metabolic system (TrueOne® 2400, ParvoMedics, Salt Lake City, UT, USA) calibrated with a known concentration of gases (20% O_2_, 8% CO_2_) and a 3 L calibration syringe, on the morning of testing. Heart rate and expired gas analysis data were recorded simultaneously on the software throughout the test. A cadence of ∼75 rpm was encouraged throughout, and the test was terminated at volitional fatigue or when cadence fell below 50 rpm.

### 2.6 Exercise intervention

Participants in the HIIT group were asked to perform four sessions per week of home‐based HIIT, involving 10 × 60 s intervals at 80%–90% peak heart rate (HR_PEAK_) on a mechanically braked arm‐crank ergometer (Monark 881E, Vansbro, Sweden). To account for changes in fitness and ensure progression, the intensity was increased by 5% every 2 weeks (i.e., 80% HR_PEAK_ for weeks 1 and 2, 85% HR_PEAK_ for weeks 3 and 4, and 90% HR_PEAK_ for weeks 5 and 6). Each exercise session included a 5‐min warm‐up and cool‐down at a low intensity (∼5 W), with 60‐s recovery intervals at ∼5 W, resulting in a total exercise time of 30 min. During each exercise training session (weeks 1–5), participants were asked to wear a chest‐worn heart rate monitor (Wahoo® Tcker X, Wahoo Fitness, Atlanta, GA, USA) and view their HR response in real‐time using a phone application. In the final week of the HIIT, heart rate data from the Actiheart™ physical activity monitor (Actiheart, Cambridge Neurotechnology Ltd, Fenstanton, UK) were used to measure compliance.

Participants were asked to avoid performing two exercise training sessions on the same day and advised to avoid performing the training sessions within 1 h of food consumption to avoid gastrointestinal issues. No other time or dietary restrictions were required for the training sessions. Participants were asked to send their heart rate data remotely to the researcher after every exercise training session (weeks 1–5) to help monitor adherence and compliance. Participants were contacted by the researcher on a weekly basis and adjustments to the exercise intensity were made if necessary.

### 2.7 Emergencies and adverse events

Participants were monitored for the following both during and after the peak aerobic capacity test and first home‐based HIIT session: chest pain, headaches, changes in vision, dizziness and light‐headedness. Blood pressure was measured immediately following the peak aerobic capacity test to identify any individuals exceeding the limits for systolic blood pressure (<85 mmHg and >200 mmHg). The laboratory has an approved procedure for emergencies, and the research team were trained in cardio‐pulmonary resuscitation. Participants were required to sign a consent form stating that they needed to be accompanied by an adult for all exercise training sessions at home. Additionally, any individuals who self‐reported regular or uncontrolled episodes of autonomic dysreflexia were asked to obtain written consent from their GP to take part in the study.

### 2.8 Outcome measures

#### 2.8.1 Aerobic capacity

Aerobic capacity (${\dot{V}}_{O_{2}\mathrm{peak}}$) was defined as the highest 15‐breath rolling average for ${\dot{V}}_{O_{2}}$. Peak power output (*P*_PEAK_) was defined as the highest power output achieved before termination of the test. Each test needed at least two of the following criteria to be deemed a valid ${\dot{V}}_{O_{2}\mathrm{peak}}$: peak HR ≥95% age‐predicted maximum for upper‐body exercise (200 bpm − age), rating of perceived exertion (RPE) ≥19, and a peak respiratory exchange ratio (RER) ≥1.10.

#### 2.8.2 Blood measurements

Fasting measures of insulin resistance, insulin sensitivity and pancreatic β‐cell function were calculated using the Homeostatic Model Assessment (HOMA‐2) calculator (Levy et al., 1998). Insulin and glucose incremental area under the curve (iAUC), and Matsuda insulin sensitivity index (ISI_Matsuda_) (Matsuda & Defronzo, 1999) were calculated to characterize responses to the OGTT. Serum and plasma samples were analysed using enzyme‐linked immunoassays (ELISA) and an automated analyser (Randox RX Daytona, Randox Laboratories, Crumlin, UK). In addition to fasting glucose and insulin, markers of inflammation (interleukin‐6, C‐reactive protein), adipokines (leptin, adiponectin), and the lipid profile (triglycerides, total cholesterol, non‐esterified fatty acids, high‐density lipoprotein cholesterol (HDL‐C), low‐density lipoprotein cholesterol (LDL‐C)) were determined.

#### 2.8.3 Physical activity energy expenditure and energy intake

Participants were asked to wear a physical activity monitor (Actiheart, Cambridge Neurotechnology Ltd, Fenstanton, UK) for 7 days before, and in the final week of the HIIT/CON period. The physical activity monitor was individually calibrated using the heart rate and corresponding energy expenditure measured during the RMR assessment and sub‐maximal exercise test, as previously described in manual wheelchair users (Nightingale et al., 2015). At least four valid days (>80% of data for that 24‐h period), including at least one weekend day, were required for the data to be included. Daily physical activity energy expenditure and time spent in different intensities of activities according to metabolic equivalents (METs) were calculated. Participants were asked to record their habitual food and fluid intake for the same 7‐day period using a set of weighing scales. Total energy intake and macronutrient composition were subsequently calculated using diet analysis software (Nutritics Ltd, Dublin, Ireland).

### 2.9 Statistical analyses

The final analysis was based on a modified intention‐to‐treat principle, whereby each participant was required to complete >75% (18/24 sessions) of planned exercise sessions to be included. Any differences in outcome measures between groups were determined using a series of ANCOVAs, with baseline values, age and time since injury entered as covariates, group allocation and sex as fixed effects, and follow‐up score as the dependent variable. Bonferroni comparisons were then performed to confirm the location of any differences, where significant interaction or main effects were observed. Any non‐normally distributed variables were log‐transformed, checked again for normality, and then used as continuous log‐transformed variables. Effect sizes (Cohen's *d*) were calculated for all variables, and interpreted as: small effect, 0.20–0.50; medium effect, 0.50–0.79; and large effect, ≥0.80. Statistical significance accepted at *P* ≤ 0.05. Data are presented as means ± 95% CI unless otherwise stated.

### 2.10 Sample size

A pilot study in individuals with SCI reported that HIIT reduced fasting insulin by 9.7 ± 7.0 mL/dL in 6 weeks (*d *= 1.10, *n* = 3) (Graham et al., 2019). To adjust for the 2:1 allocation adopted, an unequal sample size calculation was performed (www.statstodo.com/UnequalSSuze_Pgm.php). Based on an expected drop‐out of approximately 15%, and α = 0.05 and β = 0.80, we aimed to recruit a total of 40 participants in order to achieve a final sample size of 34 (23 HIIT: 11 CON).

### 2.11 Data management

All electronic files were stored on the University of Bath's shared drive, in a folder only accessible by the research team. All confidential physical data records are stored in a locked filing cabinet, in a locked room, only accessible by the corresponding author (J.B.). All serum and plasma samples were stored in a locked laboratory.

### 2.12 Trial sponsor

The trial sponsor can be contacted at: Pro-vc-research@bath.ac.uk, 01225 386141.

## 3 RESULTS

Recruitment for this study was delayed due to the COVID‐19 pandemic and study funds expired prior to completion. Consequently, a total of 31 participants were recruited for this study compared to the 40 participants stated in the protocol.

**FIGURE 2:**
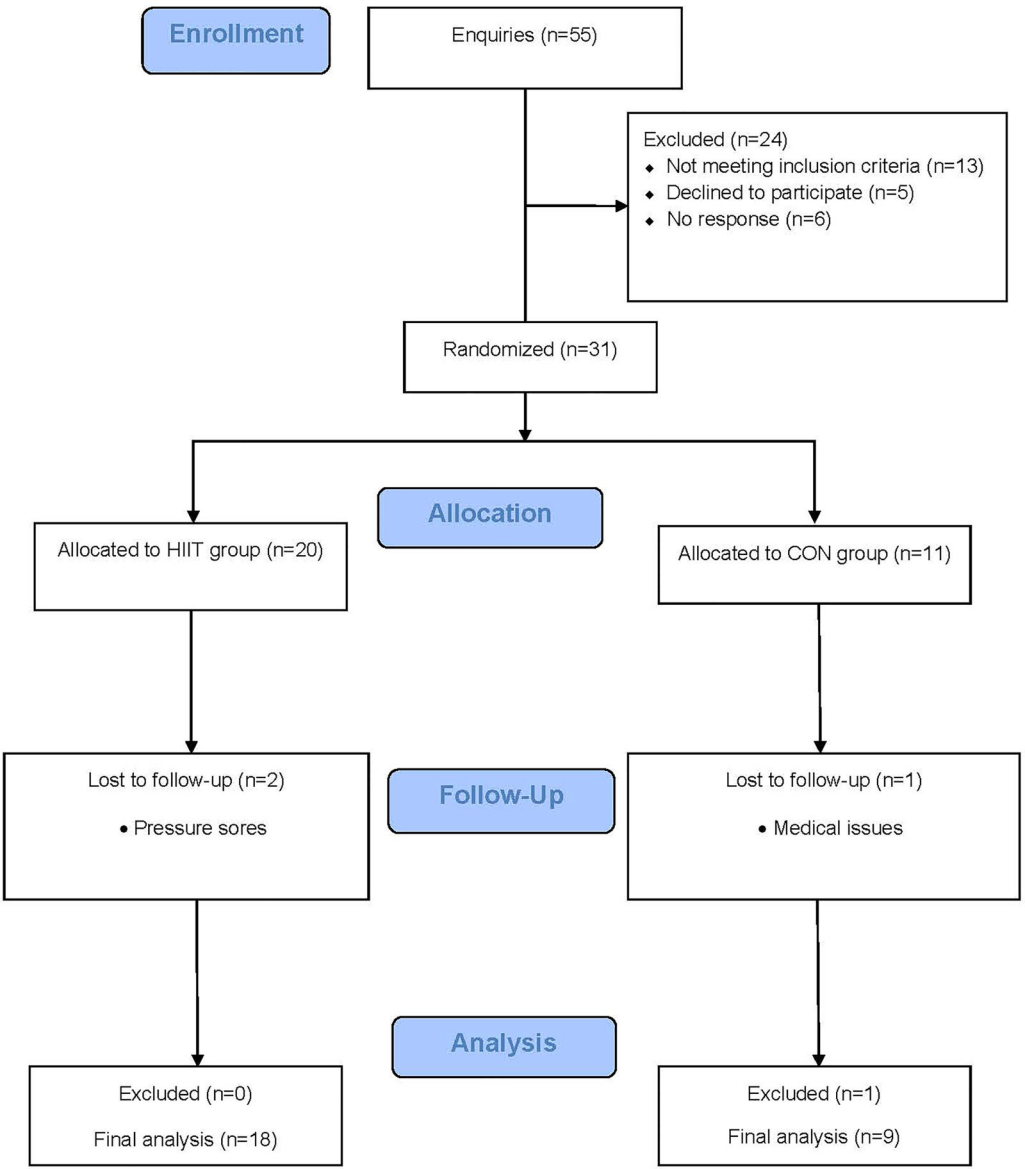
CONSORT flow diagram.

Twenty‐eight (*n* = 28) participants completed the baseline and follow‐up assessments. One participant in the CON group had a substantially higher absolute ${\dot{V}}_{O_{2}\mathrm{peak}}$ (0.98 L/min) and lower body mass (5.0 kg) at follow‐up. They were subsequently removed from all analysis due to clearly not following the guidance given to the CON group. Therefore, a total of 27 participants (18 HIIT: 9 CON) were included in the final analysis (Table 1). Three of the 31 participants who attended the initial baseline assessments withdrew from the study (Figure 2), equating to a drop‐out rate of ∼10%.

**TABLE 1:** Participant characteristics (*n* = 27).

	HIIT	CON
*n*	18	9
Age, mean ± SD (years)	46 ± 9	45 ± 7
Sex, M/F	9/9	4/5
AIS classification		
A	14	7
B	3	2
C	1	0
LOI		
Range	T3–L2	T4–12
T6 or ↑	7	3
↓T6	11	6
TSI, mean ± SD (years)	17 ± 11	12 ± 12

Abbreviations: AIS, American Spinal Cord Injury Associated Impairment Scale; LOI, level of injury; TSI, time since injury.

It was not possible to perform venepuncture (*n* = 2, both HIIT) or cannulation (*n* = 6, 3 HIIT, 3 CON) for some participants. Therefore, data for 26 participants (16 HIIT, 9 CON) are presented for all fasting blood measurements, and data for 21 participants (15 HIIT, 6 CON) for OGTT are presented. For 18 participants, IL‐6 concentrations were below the standard curve, and, therefore, this outcome measure is not included in the results.

At baseline, 24 of the participants presented with obesity (males: body fat percentage (BF%) > 22%, females: BF% > 35%) (Nash et al., 2018), 18 participants reported with low HDL‐C (males: <1.03 mmol/L, females: <1.29 mmol/L), four participants reported with hypertension (BP: ≥130/85 mmHg), six participants reported with hypertriglyceridaemia (fasting TGs: ≥1.7 mmol/L), and five participants reported with hyperglycaemia (fasting glucose: ≥5.6 mmol/L). In total, nine participants presented with cardiometabolic syndrome (obesity plus at least two of: low HDL‐C, hypertension, hypertriglyceridemia and hyperglycaemia) at baseline (Nash et al., 2018).

**FIGURE 3:**
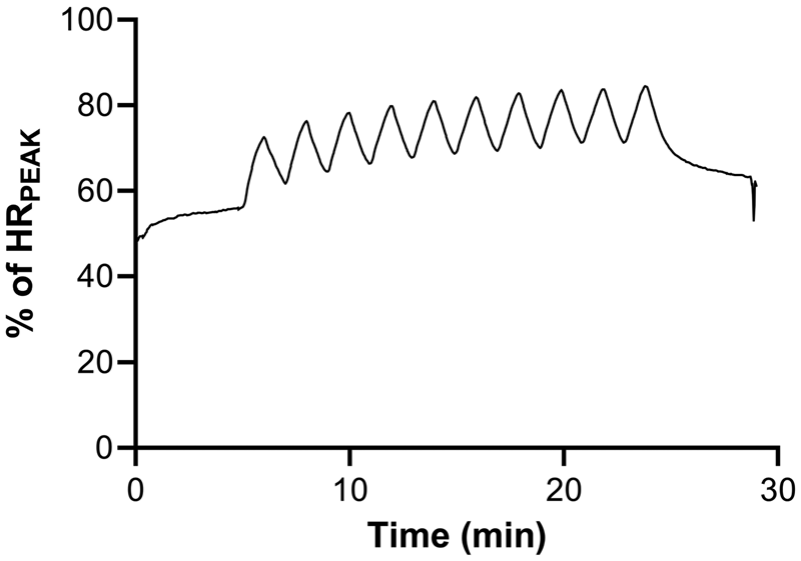
Average heart rate (expressed as %HR_PEAK_) during weeks 1–5 of HIIT.

There were no adverse events reported to the research team. All participants in the HIIT group who completed the study performed the required number of exercise sessions (18/24) to be included in the final analysis. Thirteen participants completed all sessions (100%), four participants completed 23 sessions (96%), and one participant completed 21 sessions (87.5%). The mean session RPE was 15 ± 2 for weeks 1–2, 16 ± 2 for weeks 3–4 and 17 ± 2 for weeks 5–6. Participants spent 1 ± 1 min (week 1), 3 ± 3 min (week 2), 7 ± 4 min (week 3), 7 ± 5 min (week 4), and 8 ± 5 min (week 5) at an intensity greater than 80% HR_PEAK_ (Figure 3).

### 3.1 Fasting insulin, peak power out and peak aerobic capacity

**FIGURE 4:**
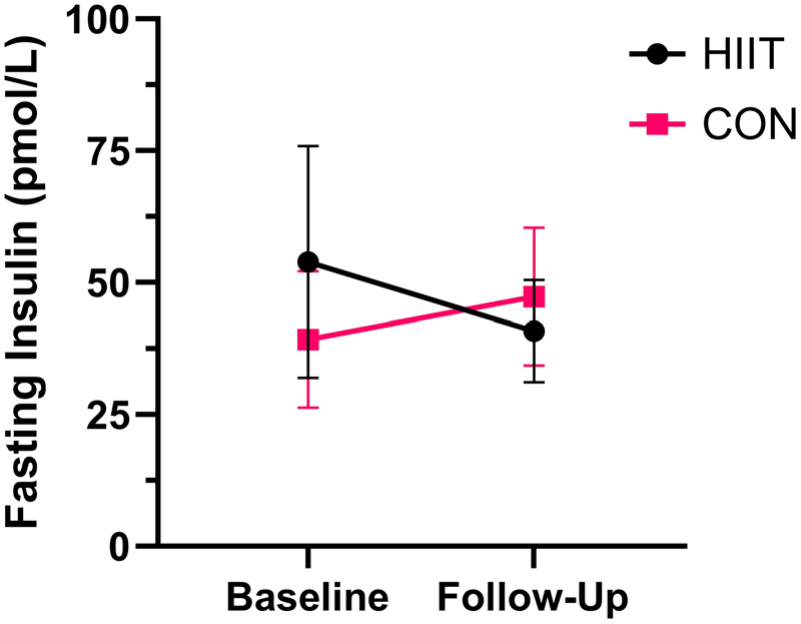
Fasting insulin at baseline and follow‐up for HIIT (*n* = 16) versus CON (*n* = 10). Follow‐up values are adjusted for covariates (baseline value, age, time since injury).

**FIGURE 5:**
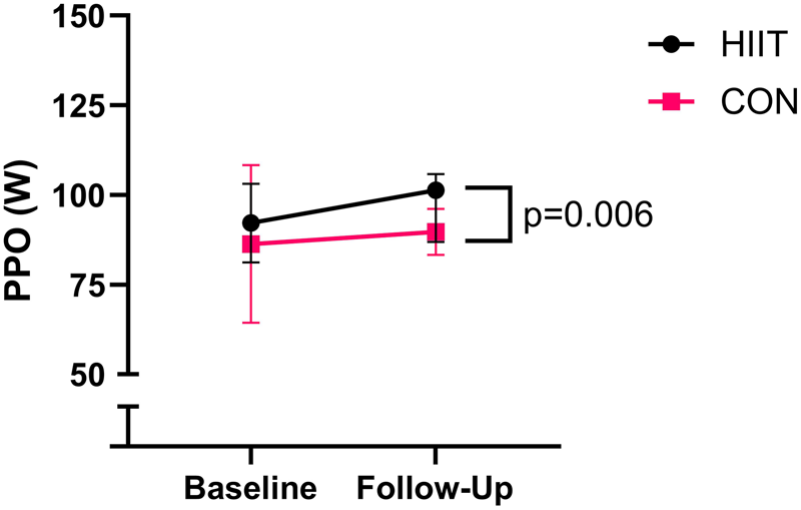
PPO at baseline and follow‐up for HIIT (*n* = 18) versus CON (*n* = 10). Follow‐up values are adjusted for covariates (baseline value, age, time since injury).

**FIGURE 6:**
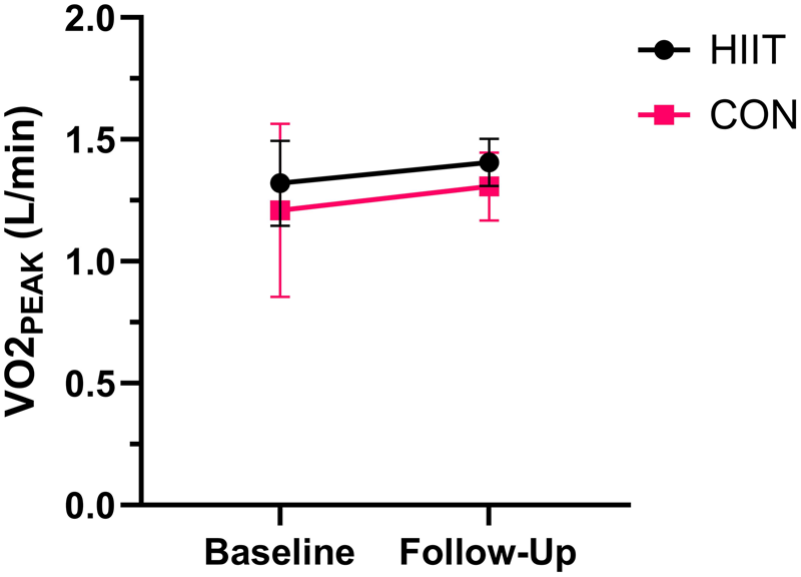
Absolute ${\dot{V}}_{O_{2}\mathrm{peak}}$ at baseline and follow‐up for HIIT (*n* = 18) versus CON (*n* = 10). Follow‐up values are adjusted for covariates (baseline value, age, time since injury).

There was no difference in fasting insulin between HIIT (*n* = 16) and CON (*n* = 9) at follow‐up (*P* = 0.415, *d *= 0.39; Figure 4). Peak power output (PPO) was higher in the HIIT (*n* = 18) compared to CON (*n* = 9) group at follow‐up (*P* = 0.006, *d *= 1.37; Figure 5). There was no difference in absolute ${\dot{V}}_{O_{2}\mathrm{peak}}$ between the HIIT (*n* = 18) and CON (*n* = 9) group at follow‐up (*P* = 0.249, *d *= 0.53; Figure 6). There was no difference in relative ${\dot{V}}_{O_{2}\mathrm{peak}}$ between HIIT (19.1 mL/kg/min, 17.9–20.3, *n* = 18) and CON (18.3 mL/kg/min, 16.5–20.0, *n* = 9) at follow‐up (*P* = 0.417, *d *= 0.37).

### 3.2 Blood biomarkers

**FIGURE 7:**
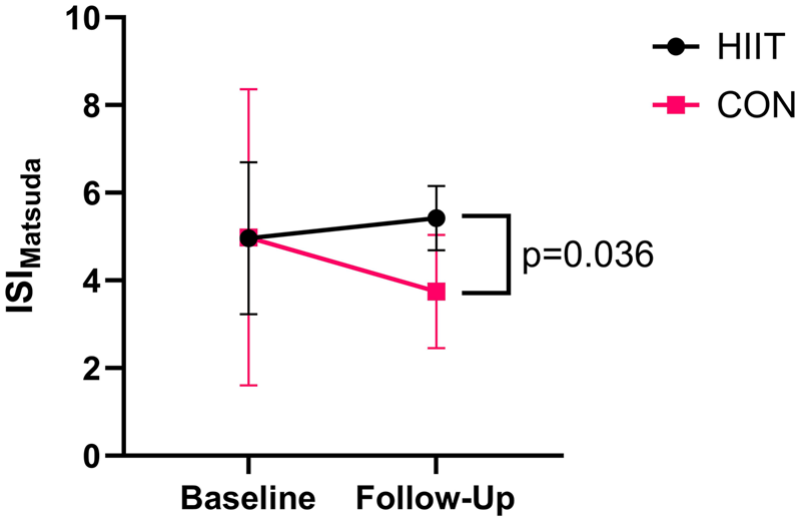
Matsuda index at baseline and follow‐up for the HIIT (*n* = 15) and CON (*n* = 6) group. Follow‐up values are adjusted for covariates (baseline value, age, time since injury).

ISI_Matsuda_ was higher at follow‐up in the HIIT group (*n* = 15) compared to the CON (*n* = 6) group (*P* = 0.036, *d *= 1.24; Figure 7). There were no differences in any other postprandial indices (Table 2), fasting blood metabolites or markers of inflammation between the HIIT and CON group at follow‐up (Table 3).

**TABLE 2:** OGTT outcomes (Data are presented as means (95% Confidence Intervals)).

	HIIT (*n* = 15)		CON (*n* = 6)			
	Baseline	Follow‐up^a^	Baseline	Follow‐up^a^	*P*	Cohen's *d*
Glucose iAUC (mmol/L × 120 min)	433 (320–545)	411 (319–503)	350 (192–508)	383 (221–545)	0.765	0.17
Glucose TAUC (mmol/L × 120 min)	1096 (859–1333)	1053 (982–1123)	908 (787–1029)	1028 (903–1153)	0.728	0.19
Insulin iAUC (nmol/L × 120 min)	44.5 (27.1–61.9)	48.1 (40.7–55.5)	69.4 (−5.4–144.3)	57.1 (44.0–70.1)	0.242	0.65
Insulin TAUC (nmol/L × 120 min)	51.2 (33.8–68.6)	53.3 (45.7–60.9)	74.9 (−1.60–151.3)	63.3 (51.0–77.7)	0.164	0.79

^a^ Follow‐up values are adjusted for covariates (baseline value, age, time since injury). *P* ≤ 0.05 considered significant. Abbreviations: iAUC, incremental area under the curve; TAUC, total area under the curve.

**TABLE 3:** Fasting blood biomarkers. (Data are presented as means (95% Confidence Intervals)).

	HIIT (*n* = 16)		CON (*n* = 9)		*P* value	Cohen's *d*
	Baseline	Follow‐up^a^	Baseline	Follow‐up^a^		
HOMA2‐IR	1.07 (0.54–1.60)	0.85 (0.70–1.00)	0.71 (0.49–0.94)	1.00 (0.80–1.21)	0.224	0.59
Glucose (mmol/L)	5.52 (4.23–6.80)	5.73 (5.27–6.19)	4.77 (4.14–5.40)	5.80 (5.19–6.41)	0.849	0.09
TGs (mmol/L)	1.13 (0.80–1.46)	1.04 (0.87–1.21)	1.20 (0.70–1.70)	1.25 (1.02–1.48)	0.142	0.72
TC (mmol/L)	5.03 (4.51–5.56)	5.47 (5.16–5.77)	5.47 (4.73–6.21)	5.13 (4.72–5.54)	0.193	0.64
HDL‐C (mmol/L)	1.12 (0.91–1.32)	1.30 (1.21–1.39)	1.27 (0.99–1.54)	1.23 (1.11–1.35)	0.329	0.47
LDL‐C (mmol/L)	3.46 (3.00–3.92)	3.71 (3.38–4.05)	3.65 (2.85–4.44)	3.33 (2.88–3.78)	0.173	0.67
NEFA (mmol/L)	0.61 (0.47–0.76)	0.56 (0.46–0.66)	0.58 (0.35–0.81)	0.55 (0.43–0.69)	0.953	0.00
Leptin (µg/L)	10.7 (6.0–15.4)	15.0 (11.3–16.9)	11.4 (6.4–16.3)	11.1 (5.3–16.9)	0.264	0.58
Adiponectin (µg/L)	9.21 (6.89–11.5)	9.58 (8.69–10.5)	10.6 (4.91–16.3)	10.1 (8.88–11.3)	0.491	0.33
CRP (mmol/L)	2.78 (1.26–4.30)	3.18 (1.45–4.92)	0.81 (0.32–1.31)	3.14 (0.76–5.53)	0.978	0.00
Lymphocytes (*n*·10^9^/L)	1.71 (1.51–1.90)	1.58 (1.45–1.70)	1.53 (1.13–1.94)	1.58 (1.41–1.75)	0.905	0.06
Neutrophils (*n*·10^9^/L)	3.64 (3.00–4.27)	3.46 (2.91–4.01)	3.40 (2.80–4.00)	3.56 (2.82–4.29)	0.835	0.09

^a^ Follow‐up values are adjusted for covariates (baseline value, age, time since injury). *P* ≤ 0.05 considered significant. Abbreviations: CRP, C‐reactive protein; HDL‐C, high‐density lipoprotein cholesterol; HOMA2‐IR, Homeostatic Model Assessment–Insulin Resistance; LDL‐C, low‐density lipoprotein cholesterol; NEFA, non‐esterified fatty acids; TC, total cholesterol; TG, triglycerides.

### 3.3 Body composition and resting blood pressure

There were no differences in body composition metrics or resting blood pressure at follow‐up between groups (Table 4).

**TABLE 4:** Body composition and resting physiological measures. (Data are presented as means (95% Confidence Intervals)).

	HIIT (*n* = 18)		CON (*n* = 9)		*P*	Cohen's *d*
	Baseline	Follow‐up^a^	Baseline	Follow‐up^a^		
Body mass (kg)	76.5 (67.7–85.3)	74.9 (74.1–75.7)	75.5 (60.8–90.2)	70.6 (59.6–81.7)	0.103	0.77
BMI (kg/m^2^)	26.6 (23.9–29.3)	26.1 (25.8–26.3)	24.6 (21.6–27.7)	25.7 (25.2–26.1)	0.115	0.72
Waist (cm)	87.7 (79.7–95.7)	85.2 (83.8–86.6)	83.8 (76.2–91.4)	84.8 (82.8–86.8)	0.778	0.13
Waist: hip	0.85 (0.81–0.89)	0.85 (0.83–0.86)	0.86 (0.81–0.92)	0.82 (0.79–0.84)	0.089	0.80
Body fat (%)	39.5 (34.7–44.2)	39.3 (38.3–40.3)	36.4 (29.9–42.9)	38.5 (37.0–40.0)	0.365	0.41
Fat mass (kg)	30.2 (24.5–36.0)	29.4 (28.4–30.3)	25.0 (19.7–30.4)	28.5 (27.1–29.8)	0.286	0.49
Fat‐free mass (kg)	42.4 (37.3–47.5)	41.8 (40.0–42.5)	41.6 (33.6–49.7)	41.7 (40.6–42.8)	0.926	0.00
Resting HR (bpm)	65 (59–70)	65 (62–67)	66 (58–73)	64 (60–67)	0.689	0.18
Systolic BP (mmHg)	125 (120–129)	118 (114–123)	114 (106–123)	117 (111–124)	0.774	0.13
Diastolic BP (mmHg)	81 (78–84)	78 (75–81)	77 (72–82)	78 (74–82)	0.942	0.00

^a^ Follow‐up values are adjusted for covariates (baseline value, age, time since injury). *P* ≤ 0.05 considered significant. Abbreviations: BMI, body mass index; BP, blood pressure; HR, heart rate.

### 3.4 Physical activity and diet

Sedentary time was higher at follow‐up in the HIIT compared to CON group (*P* = 0.040, *d *= 1.21), and light physical activity time was higher in the CON compared to HIIT group (*P* = 0.025, *d *= 1.35). There were no other differences in measures of physical activity or energy intake and macronutrient composition at follow‐up between groups (Table 5).

**TABLE 5:** Physical activity and diet measures. (Data are presented as means (95% Confidence Intervals)).

	HIIT (*n* = 18)		CON (*n* = 9)			
	Baseline	Follow‐up^a^	Baseline	Follow‐up^a^	*P*	Cohen's *d*
PAEE (kcal)^b^	471 (354–586)	418 (298–538)	327 (227–426)	572 (426–719)	0.122	0.88
Sedentary (min/day)^b^	642 (598–687)	687 (595–780)	723 (628–818)	522 (404–639)	**0.040**	1.21
Physical Activity						
Light (min/day)^b^	296 (252–340)	250 (167–333)	227 (134–320)	413 (309–518)	**0.025**	1.35
Moderate (min/day)^b^	20 (10–29)	19 (3–35)	9 (2–16)	25 (7–42)	0.661	0.24
Vigorous (min/day)^b^	0 (0–0)	0 (−1–1)	0 (0–0)	1 (0–3)	0.271	0.61
Energy intake (kcal)	1559 (1432–1686)	1529 (1426–1632)	1466 (1190–1742)	1525 (1377–1673)	0.966	0.00
Carbohydrate (g/day)	153 (128–178)	163 (147–180)	149 (117–181)	154 (131–178)	0.529	0.29
Fat (g/day)	66 (59–73)	64 (57–70)	58 (46–70)	61 (52–70)	0.621	0.22
Protein (g/day)	73 (64–82)	69 (64–74)	67 (57–76)	67 (59–75)	0.682	0.19
Alcohol (g/day)	6 (2–9)	8 (1–16)	12 (3–22)	10 (−1–21)	0.860	0.09

^a^ Follow‐up values are adjusted for covariates (baseline value, age, time since injury).

^b^ *n* = 13 for HIIT, *n* = 9 for CON. *P* ≤ 0.05 considered significant. Abbreviation: PAEE, physical activity energy expenditure.

### 3.5 Psychological measures

There were no differences in exercise self‐efficacy, shoulder pain, fatigue, subjective health and wellbeing, or functional independence between the HIIT and CON group at follow‐up (Table 6).

**TABLE 6:** Measures of health and well‐being.

	HIIT (*n* = 18)		CON (*n* = 9)		*P*	Cohen's *d*
	Baseline	Follow‐up^a^	Baseline	Follow‐up^a^		
Exercise self‐efficacy	34 (31–36)	35 (34–36)	33 (31–35)	34 (32–35)	0.414	0.38
Shoulder pain	14.5 (6.7–22.3)	15.4 (7.6–23.1)	12.3 (6.1–18.6)	17.2 (5.4–28.4)	0.787	0.13
Fatigue severity	3.7 (3.1–4.3)	3.5 (3.0–4.0)	3.9 (2.9–4.9)	4.0 (3.3–4.8)	0.254	0.52
Global fatigue	7 (6–8)	7 (6–8)	7 (5–8)	7 (6–9)	0.877	0.06
Health‐related quality of life^b^						
Physical component	61.5 (56.7–66.4)	60.0 (57.7–62.4)	56.8 (49.1–64.4)	62.2 (59.0–65.4)	0.275	0.16
Mental component	55.6 (52.0–59.2)	57.4 (54.6–60.2)	50.6 (43.2–57.9)	54.3 (50.0–58.2)	0.194	0.62
Functional independence	68 (66–70)	66 (65–68)	67 (64–71)	69 (67–71)	0.079	0.83

^a^ Follow‐up values are adjusted for covariates (baseline value, age, time since injury).

^b^ *n* = 17 for HIIT. *P* ≤ 0.05 considered significant.

## 4 DISCUSSION

The aim of this randomized controlled trial was to determine the effectiveness of upper‐body HIIT for improving cardiometabolic component risks in persons with chronic paraplegia. The primary outcome measures were fasting insulin concentrations, PPO and ${\dot{V}}_{O_{2}\mathrm{peak}}$. There were no differences in fasting insulin concentrations or ${\dot{V}}_{O_{2}\mathrm{peak}}$. At follow‐up and after adjustment for baseline values, PPO was higher in the HIIT group compared to the CON group. Additionally, ISI_Matsuda_ was higher at follow‐up in the HIIT group compared to the CON group. There were no other significant differences in any cardiometabolic component risk factors between the HIIT and CON group.

In contrast to our primary hypothesis, upper‐body HIIT had no effect on fasting insulin concentrations. This contrasts with findings from a meta‐analysis reporting that lower‐body HIIT is effective for reducing fasting insulin in the general population (Jelleyman et al., 2015). This finding is also different from SCI‐specific studies demonstrating that upper‐body MICT is effective for improving fasting insulin resistance (Kim et al., 2015; Bresnahan et al., 2019; Nightingale, Walhin et al., 2017). Our previous work demonstrated that a single bout of upper‐body HIIT (10 × 60 s intervals at 80% PPO) had no effect on next‐day fasting insulin in non‐SCI adults (Farrow et al., 2022). Combined with findings from this randomized controlled trial, it suggests that upper‐body HIIT does not provide a sufficient stimulus to improve fasting insulin sensitivity, at least within 6 weeks. It is important to note that 25 participants were included in the fasting insulin analysis, lower than the 34 participants our a priori sample size calculation stated were needed to determine a statistically significant difference between groups.

ISI_Matsuda_‐derived insulin sensitivity was higher following HIIT compared to CON. This index considers both fasting and postprandial glucose/insulin concentrations, and therefore provides an estimate of both peripheral and hepatic (i.e., whole‐body) insulin sensitivity. Two previous studies reported no difference in ISI_Matsuda_ following 6 weeks (180 min/week, 60%–65% ${\dot{V}}_{O_{2}\mathrm{peak}}$) and 10 weeks (90 min per week, 70% ${\dot{V}}_{O_{2}\mathrm{peak}}$) of upper‐body MICT despite significant reductions in fasting insulin and HOMA–insulin resistance (HOMA‐IR) (Bresnahan et al., 2019; Nightingale, Walhin et al., 2017). A recent meta‐analysis concluded that in non‐SCI individuals HIIT is effective at reducing postprandial glycaemia and insulinaemia, but only in participants with impaired glucose control (fasting glucose: ≥5.6 mmol/L or 2 h plasma glucose ≥7.8 mmol/L) (Khalafi et al., 2022). Similarly, ISI_Matsuda_ increased following HIIT in three of the four participants meeting this definition of impaired glucose control. Moderate effect sizes were observed for insulin total AUC (*d *= 0.65, *P* = 0.242) and iAUC (*d *= 0.79, *P* = 0.164), and this may explain the significant difference in ISI_Matsuda_. Importantly, when the raw uncorrected data are examined, ISI_Matsuda_ increased by 10.3 ± 29.7% in the HIIT group and decreased by 12.6 ± 24.8% in the CON group, suggesting that our intervention offset deleterious changes in insulin sensitivity.

PPO increased by 12% following HIIT and remained almost unchanged in the CON group (+1%). This increase in physical capacity is consistent with previous upper‐body HIIT interventions in individuals with SCI (McLeod et al., 2020; Tordi et al., 2001). Importantly, improvements in physical capacity are likely to make activities of daily living easier to perform and have been associated with increased life satisfaction in persons with SCI (Manns & Chad, 1999). The magnitude of the increase in PPO is lower than the 20% increase reported following 6 weeks of MICT (180 min/week, 60%–65% ${\dot{V}}_{O_{2}\mathrm{peak}}$) (Nightingale, Walhin et al., 2017). However, the HIIT intervention in the present study involved just 40 min per week of ‘active’ exercise, and therefore, a 12% increase in PPO represents a substantial improvement in this context. Alternatively, the modest improvements in PPO may be due to the baseline fitness levels of our participants. Twenty of the 27 participants to complete the study had an above average baseline ${\dot{V}}_{O_{2}\mathrm{peak}}$ (>17.69 mL/kg/min for males, >13.2 mL/kg/min for females) (Simmons et al., 2014). Surprisingly, the increase in PPO following HIIT did not translate to any change in either absolute or relative ${\dot{V}}_{O_{2}\mathrm{peak}}$, despite a moderate correlation (*P* = 0.038, *r* = 0.49) between the change in PPO and absolute ${\dot{V}}_{O_{2}\mathrm{peak}}$.

There were no other changes in any other cardiometabolic risk factors measured. In non‐SCI populations, meta‐analyses have reported improvements following HIIT in a variety of measures including adiponectin and leptin (Khalifi & Symonds, 2020), systolic and diastolic BP (Campbell et al., 2019), and HDL‐C (Wood et al., 2019). There are several factors that may explain the lack of changes observed in the present study, including the duration of intervention, time spent exercising at a ‘high’ intensity, and physical activity substitution. Firstly, it is possible that 6 weeks is insufficient to induce adaptation to upper‐body HIIT. A meta‐analysis reported that the adaptations to short‐term HIIT (<12 weeks: ${\dot{V}}_{O_{2}\mathrm{peak}}$, diastolic blood pressure, fasting glucose) differ from long‐term HIIT (≥12 weeks: ${\dot{V}}_{O_{2}\mathrm{peak}}$, waist circumference, BF%, systolic blood pressure, diastolic blood pressure, resting HR) (Batacan et al., 2017).

Secondly, it is possible that the time spent exercising ≥80% HR_PEAK_ was insufficient. Figure 3 highlights that it took three to four high‐intensity intervals to reach the target HR. This is likely due to the short duration of the high‐intensity interval efforts and has been observed in cycling‐based studies (Currie et al., 2013). Therefore, it could be prudent to either extend the duration (8 × 2 min intervals) or number (e.g., 12–15 × 1 min intervals) of high‐intensity intervals to increase the time spent exercising ≥80% HR_PEAK_. Further to this, there was also considerable inter‐individual variability in HR responses across the HIIT intervention, with three participants self‐reporting that they struggled to reach the target HR. For example, one participant (T6 injury) performed just 1 ± 1 min of each session (averaged across weeks 1–5) at an intensity meeting the definition of HIIT (≥80% HR_PEAK_). However, they reported an RPE of 18 ± 0 across the intervention suggesting they were working very hard. Conversely, one participant (L2 injury) performed 6 ± 5 min of each session at an intensity ≥80% HR_PEAK_ but reported an RPE of 13 ± 1 across the intervention. This also highlights the methodological issues of using %HR_PEAK_ to prescribe exercise intensity in individuals with a SCI, as recently raised by Hutchinson & Goosey‐Tolfrey (2022). Specifically, for the same %HR_PEAK_, the intensity domain (defined using lactate thresholds) can vary substantially between individuals with SCI. This variation between intensity domains is not present when exercise intensity is prescribed using RPE, suggesting RPE‐based HIIT programmes may provide more homogeneous training responses across participants with an SCI. Future studies may wish to incorporate the clinical population guidelines (Taylor et al., 2019), which combine RPE and %HR_PEAK_ in the prescription of HIIT.

Thirdly, it appears that individuals in the HIIT group may have ‘substituted’ the prescribed exercise in place of their habitual non‐prescribed physical activity (Thompson et al., 2014). This is evidenced by our objectively measured physical activity data, with individuals in the HIIT group performing 15 min/day more sedentary time (<1.5 METs) and 18 min/day less light physical activity (1.5–3.0 METs) at follow‐up compared to baseline. Comparatively, individuals in the CON group performed substantially less sedentary time (171 min/day) and more light physical activity (161 min/day) at follow‐up compared to baseline. This ‘control group’ response to physical activity interventions has been widely reported (Waters et al., 2012), and the changes in physical activity behaviours for both groups may have confounded our results. Future studies in this area and population should consider utilizing a control lead‐in phase, to enable all participants to take part in the intervention.

Finally, but perhaps most likely, the nature of upper‐body exercise, involving only a small volume of active skeletal muscle mass and thus limited scope to effect whole‐body biomarkers, may be responsible for these results. Compared to whole‐ or lower‐body exercise at the same relative exercise intensity, upper‐body exercise elicits a lower cardiovascular strain, energy expenditure and metabolic disturbance (Nightingale, Metcalfe, Vollaard & Bilzon, 2017). Four randomized controlled trials have now demonstrated that upper‐body exercise interventions fail to elicit benefits to traditional cardiometabolic component risk factors in individuals with SCI (Alrashidi et al., 2021; Hansen et al., 2023; Nightingale, Walhin et al., 2017). Given the challenges in performing such studies in this population and to develop knowledge more efficiently, researchers may wish to consider diverting attention to multi‐modal exercise interventions with upper‐body resistance training and lower‐body components (e.g., low‐force electrical stimulation) (Sanchez et al., 2023). These modalities may be realistic and achievable options for community‐dwelling individuals and appear to show some promise in modulating cardiometabolic component risk factors (Mogharnasi et al., 2019; Petrie et al., 2022).

Despite the lack of changes in cardiometabolic risk factors, this study supports findings from a previous pilot study that upper‐body HIIT is safe and feasible for individuals with chronic paraplegia (Koontz et al., 2021). There were two dropouts in the HIIT group due to pressure sores unrelated to the intervention. Importantly, there were no adverse events reported across 425 home‐based exercise sessions, and compliance was very high (98%). This alleviates some concerns of the real‐world applicability of upper‐body HIIT (Biddle & Batterham, 2015). The compliance rate was likely high due to the home‐based nature of the exercise intervention, which addresses some of the barriers to exercise participation in this population (e.g., transportation, lack of equipment/facilities) (Kehn & Kroll, 2009). The weekly check‐ins with the research team, and use of live feedback (i.e., Wahoo Tcker X) are all likely to have improved exercise compliance.

### 4.1 Conclusion

This randomized controlled trial demonstrated that home‐based arm crank ergometry HIIT is safe and feasible for individuals with chronic paraplegia. The 6‐week HIIT intervention improved upper‐body peak power output and postprandial insulin sensitivity. There were no other beneficial effects on a wide range of cardiometabolic component risk factors.

## AUTHOR CONTRIBUTIONS

The results of the present study are presented clearly, honestly and without fabrication, falsification, or inappropriate data manipulation. Matthew Farrow, Jennifer Maher, Sean Williams, Dylan Thompson, and James Bilzon were responsible for study conception and design. Data collection and analysis were performed by Matthew Farrow, Jennifer Maher, Rachel Deere, and Bruno Spellanzon. Matthew Farrow drafted the manuscript. All authors critically revised the manuscript for important intellectual content. All authors have read and approved the final version of this manuscript and agree to be accountable for all aspects of the work in ensuring that questions related to the accuracy or integrity of any part of the work are appropriately investigated and resolved. All persons designated as authors qualify for authorship, and all those who qualify for authorship are listed.

## CONFLICT OF INTEREST

The authors of this manuscript declare no conflicts of interest.

## Data Availability

The raw and anonymized data presented in this study are available on the University of Bath Research Data Archive (https://doi.org/10.15125/BATH‐01385).
